# Comparison of Human Social Brain Activity During Eye-Contact With Another Human and a Humanoid Robot

**DOI:** 10.3389/frobt.2020.599581

**Published:** 2021-01-29

**Authors:** Megan S. Kelley, J. Adam Noah, Xian Zhang, Brian Scassellati, Joy Hirsch

**Affiliations:** ^1^ Interdepartmental Neuroscience Program, Yale School of Medicine, New Haven, CT, United States; ^2^ Brain Function Laboratory, Department of Psychiatry, Yale School of Medicine, New Haven, CT, United States; ^3^ Social Robotics Laboratory, Department of Computer Science, Yale University, New Haven, CT, United States; ^4^ Departments of Neuroscience and Comparative Medicine, Yale School of Medicine, New Haven, CT, United States; ^5^ Department of Medical Physics and Biomedical Engineering, University College London, London, United Kingdom

**Keywords:** social cognition, fNIRS, human-robot interaction, eye-contact, tempoparietal junction, social engagement, dorsolateral prefontal cortex, TPJ

## Abstract

Robot design to simulate interpersonal social interaction is an active area of research with applications in therapy and companionship. Neural responses to eye-to-eye contact in humans have recently been employed to determine the neural systems that are active during social interactions. Whether eye-contact with a social robot engages the same neural system remains to be seen. Here, we employ a similar approach to compare human-human and human-robot social interactions. We assume that if human-human and human-robot eye-contact elicit similar neural activity in the human, then the perceptual and cognitive processing is also the same for human and robot. That is, the robot is processed similar to the human. However, if neural effects are different, then perceptual and cognitive processing is assumed to be different. In this study neural activity was compared for human-to-human and human-to-robot conditions using near infrared spectroscopy for neural imaging, and a robot (Maki) with eyes that blink and move right and left. Eye-contact was confirmed by eye-tracking for both conditions. Increased neural activity was observed in human social systems including the right temporal parietal junction and the dorsolateral prefrontal cortex during human-human eye contact but not human-robot eye-contact. This suggests that the type of human-robot eye-contact used here is not sufficient to engage the right temporoparietal junction in the human. This study establishes a foundation for future research into human-robot eye-contact to determine how elements of robot design and behavior impact human social processing within this type of interaction and may offer a method for capturing difficult to quantify components of human-robot interaction, such as social engagement.

## Introduction

In the era of social distancing, the importance of direct social interaction to personal health and well-being has never been more clear (Brooke and Jackson, 2020; Okruszek et al., 2020). Fields such as social robotics take innovative approaches to addressing this concern by utilizing animatronics and artificial intelligence to create simulations of social interaction such as that found in human-human interaction (Belpaeme et al., 2018; Scassellati, et al., 2018a) or human-pet companionship (Wada et al., 2010; Kawaguchi et al., 2012; Shibata, 2012). With our experiences and norms of social interaction changing in response to the COVID-19 pandemic, the innovation, expansion, and integration of such tools into society are in demand. Scientific advancements have enabled development of robots which can accurately recognize and respond to the world around it. One approach to simulation of social interaction between humans and robots requires a grasp of how the elements of robot design influence the neurological systems of the human brain involved in recognizing and processing direct social interaction, and how that processing compares to that during human-human interaction (Cross et al., 2019; Henschel et al., 2020; Wykowska, 2020).

The human brain is distinctly sensitive to social cues (Di Paolo and De Jaegher, 2012; Powell et al., 2018) and many of the roles that robots could potentially fill require not only the recognition and production of subtle social cues on the part of the robot, but also recognition from humans of the robot’s social cues as valid. The success of human-human interaction hinges on a mix of verbal and non-verbal behavior which conveys information to others and influences their internal state, and in some situations robots will be required to engage in equally complex ways. Eye-to-eye contact is just one example of a behavior that carries robust social implications which impact the outcome of an interaction via changes to the emotional internal state of those engaged in it (Kleinke, 1986). It is also an active area of research for human-robot interaction (Admoni and Scassellati, 2017) which has been shown to meaningfully impact robot engagement (Kompatsiari et al., 2019). Assessing neural processing during eye-contact is one way to understand human perception of robot social behavior, as this complex internal cascade is, anecdotally, ineffable and thus difficult to capture via questionnaires. Understanding how a particular robot in a particular situation impacts the neural processing of those who interact with it, as compared to another human in the same role, will enable tailoring of robots to fulfill roles which may benefit from having some but not all elements of human-human interaction (Disalvo et al., 2002; Sciutti et al., 2012a). An additional benefit of this kind of inquiry can be gained for cognitive neuroscience. Robots occupy a unique space on the spectrum from object to agent which can be manipulated through robot behavior, perception, and reaction, as well as the development of expectations and beliefs regarding the robot within the human interacting partner. Due to these qualities, robots are a valuable tool for parsing elements of human neural processing and comparison of the processing of humans and of robots as social partners is a fruitful ground for discovery (Rauchbauer et al., 2019; Sciutti et al., 2012a).

The importance of such understanding for the development of artificial social intelligence has long been recognized (Dautenhahn, 1997). However, due to technological limitations on the collection of neural activity during direct interaction, little is known about the neurological processing of the human brain during human-robot interaction as compared to human-human interaction. Much of what is known has been primarily acquired via functional magnetic resonance imaging (fMRI) (Gazzola et al., 2007; Hegel et al., 2008; Chaminade et al., 2012; Sciutti et al., 2012b; Özdem et al., 2017; Rauchbauer et al., 2019), but this technique imposes many practical limitations on ecological validity. The development of functional Near Infrared Spectroscopy (fNIRS) has made such data collection accessible. FNIRS uses absorption of near-infrared light to measure hemodynamic oxyhemoglobin (OxyHb) and deoxyhemoglobin (deOxyHb) concentrations in the cortex as a proxy for neural activity (Jobsis, 1977; Ferrari and Quaresima, 2012; Scholkmann et al., 2014). Advances in acquisition and developments in signal processing and methods have made fNIRS a viable alternative to fMRI for investigating adult perception and cognition, particularly in natural conditions. Techniques for representation of fNIRS signals in three-dimensional space allows for easy comparison with those acquired using fMRI (Noah et al., 2015; Nguyen et al., 2016; Nguyen and Hong, 2016).

Presented here are the results of a study to demonstrate how fNIRS applied to human-robot interaction and human-human interaction can be used to make inferences about human social brain functioning as well as efficacy of social robot design. This study utilized fNIRS during an established eye-contact paradigm to compare neurological processing of human-human and human-robot interaction. Participants engaged in periods of structured eye-contact with another human and with “Maki”, a simplistic human-like robot head (Payne, 2018; Scassellati et al., 2018b). It was hypothesized that participants would show significant rTPJ activity when making eye-contact with their human partner but not their robot partner. In this context, we assume that there is a direct correspondence of neural activity to perception and cognitive processing. Therefore, if eye contact with a human and with a robot elicit similar neural activity in sociocognitive regions, then it can be assumed that the cognitive behavior is also the same. If, however, the neural effects of a human partner and a robot partner are different, then we can conclude that sociocognitive processing is also different. Such differences might occur even when apparent behavior seems the same.

The right temporoparietal junction (rTPJ) was chosen as it is a processing hub for social cognition (Carter & Huettel, 2013) which is believed to be involved in reasoning about the internal mental state of others, referred to as theory of mind (ToM) (Molenberghs et al., 2016; Premack and Woodruff, 1978; Saxe and Kanwisher, 2003; Saxe, 2010). Not only has past research shown that the rTPJ is involved in explicit ToM (Sommer et al., 2007; Aichhorn et al., 2008), it is also spontaneously engaged during tasks with implicit ToM implications (Molenberghs et al., 2016; Dravida et al., 2020; Richardson and Saxe, 2020). It is active during direct human eye-to-eye contact at a level much higher than that found during human picture or video eye-contact (Hirsch et al., 2017; Noah et al., 2020). This is true even in the absence of explicit sociocognitive task demands. What these past studies suggest is that: 1) human-human eye-contact has a uniquely strong impact on activity in the rTPJ; 2) spontaneous involvement of the rTPJ suggests individuals engage in implicit ToM during human-human eye-contact; and 3) appearance, movement, and coordinated task behavior—those things shared between a real person and a simulation of a person—are not sufficient to engage this region comparably to direct human-human interaction. These highly replicable findings suggests that studying the human brain during eye-contact with a robot may give unique insight into the successes and failures of social robot design based on the patterns of activity seen and their similarity or dissimilarity to that of the processing of humans. Additionally, assessing human-robot eye-contact will shed light on how the rTPJ is impacted by characteristics which are shared by a human and a robot, but which are not captured by traditional controls like pictures or videos of people.

## Methods and Materials

### Participants

Fifteen healthy adults (66% female; 66% white; mean age of 30.1 ± 12.2; 93% right-handed; (Oldfield, 1971)) participated in the study. All participants provided written informed consent in accordance with guidelines approved by the Yale University Human Investigation Committee (HIC #1501015178) and were reimbursed for participation.

### Experimental Eye-Contact Procedure

**FIGURE 1:**
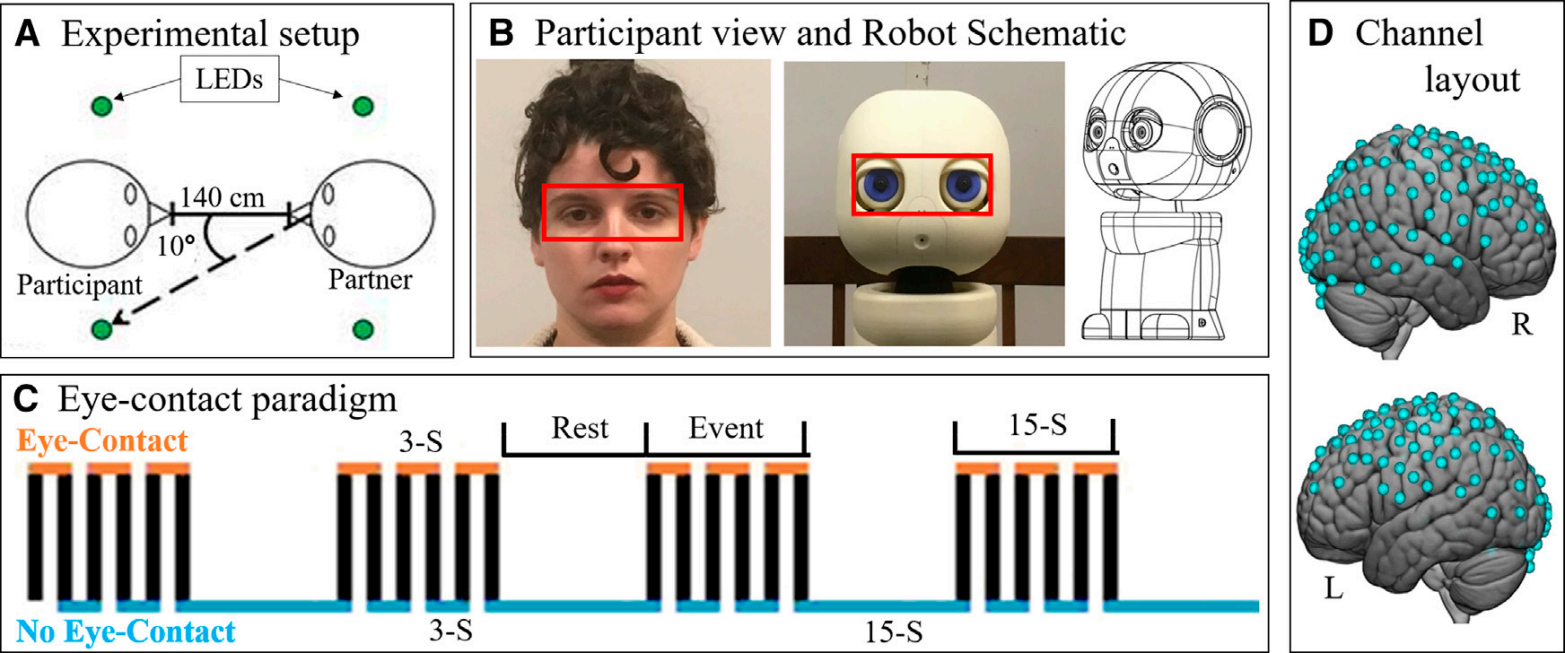
Paradigm. **(A)** Schematic (view from above) showing the experimental setup. Participants were seated approximately 140 cm from their partner. On either side of the participant's and the partner's head were LEDs (green circles). Participants (n = 15) alternated between looking into the eyes of their partner and diverting their gaze 10° to one of the lights. **(B)** Participants completed the eye-contact task with two partners: a human **(left)** and a robot (patient view: center, schematic: right). Red boxes was used to analyze eye-tracking data in order to assess task compliance. Eye boxes took up approximately the same area of participant visual angle. Schematic source: HelloRobo, Atlanta, Georgia. **(C)** Paradigm timeline for a single run. Each run consisted of alternating 15-second (S) event and rest blocks. Event blocks consisted of alternating 3-S periods of eye contact (orange) and no eye contact (blue). Rest blocks consisted of no eye contact. **(D)** Hemodynamic data was collected during the task. This included 134 channels spread across both hemispheres of the cortex via 80 pairs of optodes placed against the scalp. Blue dots represent points of data collation relative to the cortex.

Participants completed an eye-contact task. See Figure 1A for a schematic of the room layout during the task. The eye-contact task used in this experiment is similar to previously published studies (Hirsch et al., 2017; Noah et al., 2020). The task was completed while the participant was seated at a table facing a partner—either the human or the robot (Figure 1B)—at a distance of 140 cm. At the start of each task, an auditory cue prompted participants to gaze at the eyes of their partner. Subsequent auditory tones cued eye gaze to alternatingly move to one of two light emitting diodes (LEDs) or back to the partner’s eyes, according to the protocol time series (Figure 1C). Each task run consisted of six 30 s epochs, with one epoch including one 15 s event block and one 15 s rest block, for a total run length of 3 min. Event blocks consisted of alternating 3 s periods of gaze at partners eyes and gaze at an LED. Rest blocks consisted of gaze at an LED.

Every participant completed the task twice each with two partners: another real human—a confederate employed by the lab—and a robot bust (Figure 1B). The order of partners was counter balanced. In both partner conditions, the partner completed the eye-contact task concurrently with the participant, such that the participant and the current partner had gaze diverted to an LED and gaze towards partner at the same time. The timing and range of motion of participant eye movements were comparable for both partners. This task was completed twice with each partner, for a total of four runs. During the task, hemodynamic and behavioral data were collected using functional Near Infrared Spectroscopy (fNIRS) and desk mounted eye-tracking.

### Robot Partner

The robot partner was a 3D-printed humanoid bust named Maki (HelloRobo, Atlanta, Georgia) (Payne, 2018; Scassellati et al., 2018b) which can simulate human eye-movements but which otherwise lacks human features, movements, or reactive capabilities. Maki’s eyes were designed to have comparable components to that of human eyes: whites surrounding a colored iris with a black “pupil” in the center (Figure 1B). Maki’s movements were carried out using an Arduino IDE controlling six servo motors, giving it six degrees of freedom: head turn left-right and tilt up-down; left eye move left-right and up-down; right eye move left-right and up-down; and eye-lids open-close. This robot was chosen due to the design emphasis on eye movements, as well as its simplified similarity to the overall size and organization of the human face which was hypothesized to be sufficient to control for the appearance of a face (Haxby et al., 2000; Ishai, 2008; Powell et al., 2018).

Participants were introduced to each partner immediately prior to the start of the experiment. For Maki’s introduction, it was initially positioned such that its eyes were closed, and its head was down. Participants then witnessed the robot being turned on, at which point Maki opened its eyes, positioned its head in a neutral, straightforward position, and began blinking in a naturalistic pattern (Bentivoglio et al., 1997). Participants then watched as Maki carried out eye-movement behaviors akin to those performed in the task. This was done to minimize any neural effects related to novelty or surprise at Maki’s movements. Participants introduction to Maki consisted of telling them its name and indicating that it would be performing the task along with them. No additional abilities were insinuated or addressed. Introduction to the human partner involved them walking into the room, sitting across from the partner and introducing themselves. Dialogue between the two was neither encouraged nor discouraged. This approach to introduction was chosen to avoid giving participants any preconceptions about either the robot or human partner.

The robot completed the task via pre-coded movements timed to appear as if it was performing the task concurrently with the participant. Robot behaviors during the task were triggered via PsychoPy. The robot partner shifted its gaze position between partner face and light at timed intervals which matched the task design such that the robot appeared to look towards and away from the participant at the same time that the participant was instructed to look towards or away from the robot. The robot was not designed to nor capable of replicating all naturalistic human eye-behavior. While it can simulate general eye movements, fast behaviors like saccades and subtle movements like pupil size changes are outside of its abilities. Additionally, the robot did not perform gaze fixations due to lack of any visual feedback or input. In order to simulate a naturalistic processing delay found in humans, a 300-ms delay was incorporated between the audible cue to shift gaze position and the robot shifting its gaze. The robot also engaged in a randomized naturalistic blinking pattern.

### FNIRS Data Acquisition, Signal Processing and Data Analysis

Functional NIRS signal acquisition, optode localization, and signal processing, including global mean removal, were similar to methods described previously (Dravida et al., 2018; Dravida et al., 2020; Hirsch et al., 2017; Noah et al., 2015; Noah et al., 2020; Piva et al., 2017; Zhang et al., 2016; Zhang et al., 2017) and are summarized below. Hemodynamic signals were acquired using an 80-fiber multichannel, continuous-wave fNIRS system (LABNIRS, Shimadzu Corporation, Kyoto, Japan). Each participant was fitted with an optode cap with predefined channel distances, determined based on head circumference: large was 60 cm circumference; medium was 56.5 cm; and small was 54.5 cm. Optode distances of 3 cm were designed for the 60 cm cap layout and were scaled linearly to smaller caps. A lighted fiber-optic probe (Daiso, Hiroshima, Japan) was used to remove hair from the optode holders prior to optode placement, in order to ensure optode contact with the scalp. Optodes consisting of 40 emitter-detector pairs were arranged in a matrix across the scalp of the participant for an acquisition of 134 channels per subject (Figure 1D **)**. This extent of fNIRS data collection has never been applied to human-robot interaction. For consistency, placement of the most anterior optode-holder of the cap was centered 1 cm above the nasion.

To assure acceptable signal-to-noise ratios, attenuation of light was measured for each channel prior to the experiment, and adjustments were made as needed until all recording optodes were calibrated and able to sense known quantities of light from each wavelength (Tachibana et al., 2011; Ono et al., 2014; Noah et al., 2015). After completion of the tasks, anatomical locations of optodes in relation to standard head landmarks were determined for each participant using a Patriot 3D Digitizer (Polhemus, Colchester, VT, USA) (Okamoto and Dan, 2005; Eggebrecht et al., 2012).

Shimadzu LABNIRS systems utilize laser diodes at three wavelengths of light (780 nm, 805 nm, 830 nm). Raw optical density variations were translated into changes in relative chromophore concentrations using a Beer-Lambert equation (Hazeki and Tamura, 1988; Matcher et al., 1995). Signals were recorded at 30 Hz. Baseline drift was removed using wavelet detrending provided in NIRS-SPM (Ye et al., 2009). Subsequent analyses were performed using Matlab 2019a. Global components attributable to blood pressure and other systemic effects (Tachtsidis and Scholkmann, 2016) were removed using a principal component analysis spatial global-component filter (Zhang et al., 2016; Zhang et al., 2017) prior to general linear model (GLM) analysis (Friston et al., 1994). Comparisons between conditions were based on GLM procedures using the NIRS-SPM software package (Ye et al., 2009). Event and rest epochs within the time series (Figure 1D) were convolved with the canonical hemodynamic response function provided in SPM8 (Penny et al., 2011) and were fit to the data, providing individual ‘‘beta values’’ for each channel per participant across conditions. Montreal Neurological Institute (MNI) coordinates (Mazziotta et al., 1995) for each channel were obtained using NIRS-SPM software (Ye et al., 2009) and the 3-D coordinates obtained using the Polhemus patriot digitizer.

Once in normalized space, the individual channel-wise beta values were projected into voxel-wise space. Group results of voxel-wise t-scores based on these ‘‘beta values’’ were rendered on a standard MNI brain template. All analyses were performed on the combined OxyHb + deOxyHb signals (Silva et al., 2000; Dravida et al., 2018). This was calculated by adding the absolute value of the change in concentration of OxyHb and deOxyHb together. This combined signal is used as it reflects through a single value the stereotypical task-related anti-correlation between increase in OxyHb and decrease in deOxyHb (Seo et al., 2012). As such, the combined signal provides a more accurate reflection of task related activity by incorporating both components of the expected signal change as opposed to just one or the other.

For each condition—human partner and robot partner—task related activity was determined by contrasting voxel-wise activity during task blocks with that during rest blocks which identifies regions which are more activated during eye-contact than baseline. The resulting single condition results for human eye-contact and robot eye-contact were contrasted with each other to identify regions which showed more activity when making eye-contact with one partner or the other. A region of interest analysis was performed on the rTPJ using a mask from a previously published study (Noah et al., 2020). Effect size (classic Cohen’s *d*) was calculated using ROI beta values for all subjects to calculate mean and pooled standard deviation. Whole cortex analyses were also performed, as an exploratory analysis.

### Eye-Tracking Acquisition

Participant eye-movements during the experiment were recorded using a desk-mounted Tobii X3-120 (Stockholm, Sweden) eye-tracking system. The eye-tracker recorded eye behavior at 120 Hz. The reference scene video was recorded at 30 Hz from a scene camera positioned directly behind and above the participants’ head at 1280 x 720 pixels using a Logitech c920 camera (Lausanne, Switzerland). Eye-tracking data was analyzed using an “eye-box” which was determined via the anatomical layout of the partners face (Figure 1B, red boxes). When the eyes of the participant fell within the bounds of the eye-box of their partner, it was considered a “hit”. To assess results, the task time was divided into 3 s increments, which reflects the length of a single-eye-contact period. The percentage of frames with a hit were calculated for every 3 s period of the task. Data points in which at least one eye was not detected (due to technical difficulties or eye-blinks) were considered invalid and excluded from this calculation. Datasets which resulted in more than 1/3 of data points being invalid due to these dropped signals, blinks, or noise were excluded from analyses. Due to collection error, researcher error, or technical difficulties, 17 of 60 eye-tracking datasets were excluded. These were equally distributed between the two trial types (9 from human and 8 from robot) and therefore do not differentially impact the two conditions. If the participant complied with the task, then the proportion of eye-box hits per increment should reflect a pattern similar to that shown in Figure 1D, where eye-contact periods show a high proportion of hits and no eye-contact periods show few or no hits.

## Results

### Behavioral Eye-Tracking Results

**FIGURE 2:**
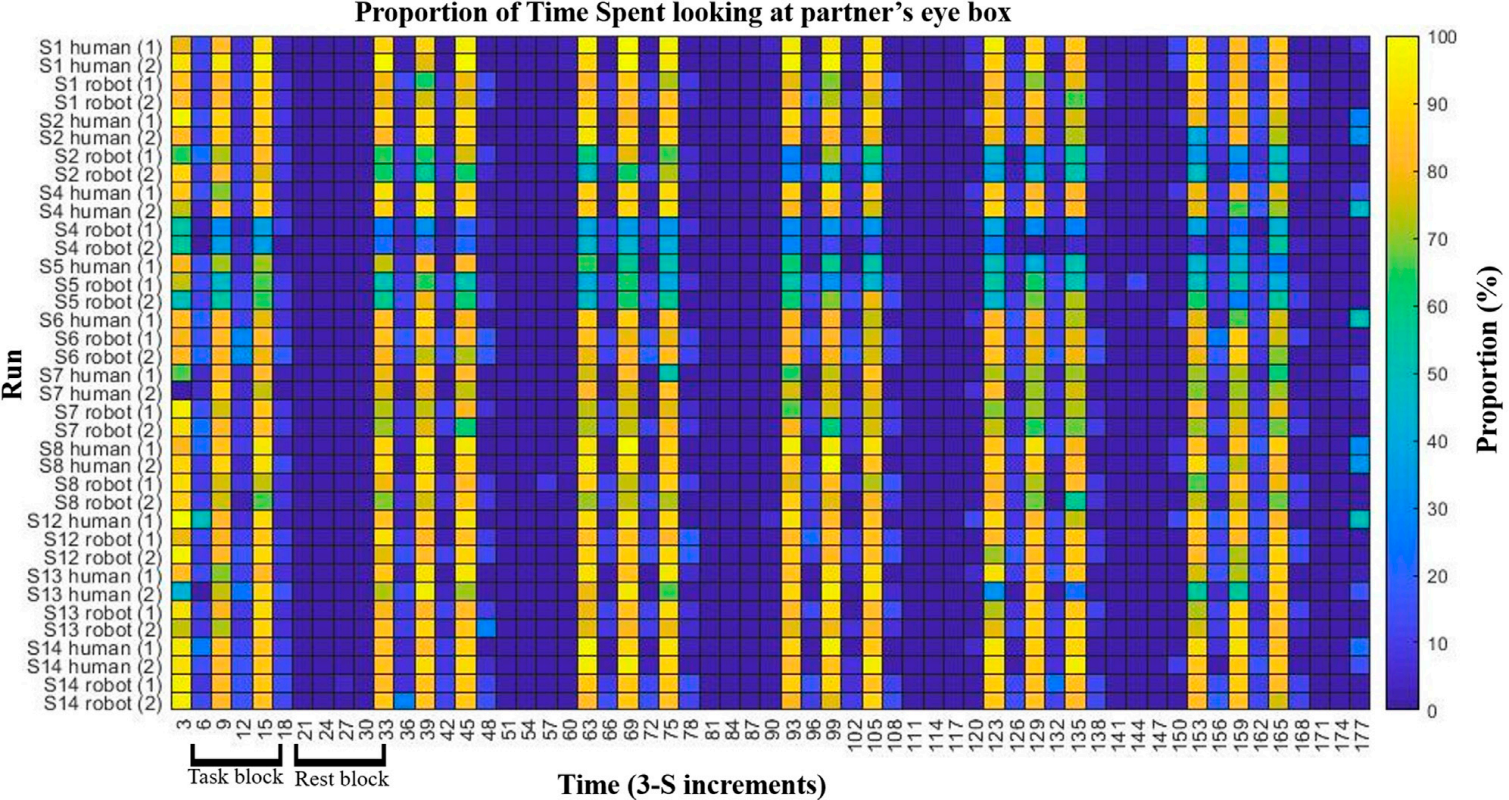
Eye tracking and task compliance. Behavioral results are shown indicating task compliance for both partners. The task bar was divided into 3 s increments (*x*-axis) and the proportion of hits on the partner’s eye box were calculated per increment. Subjects which had at least one valid eye-tracking data set per condition are shown (*y*-axis). Color indicates the proportion of the 3 seconds spent in the partners eye-box, after excluding data points in which one or more eyes were not recorded (e.g., blinks). Subjects showed more eye-box hits during the eye-contact periods (every other increment during task blocks) then during no eye contact periods (remaining task blocs increments as well as all rest block increments). Two subjects, S02 and S04, appear to have made less eye-contact with the robot than with the human partner. In order to ensure that this did not confound results, hemodynamic analyses were performed with both the inclusion and exclusion of those two subjects.

**FIGURE 3:**
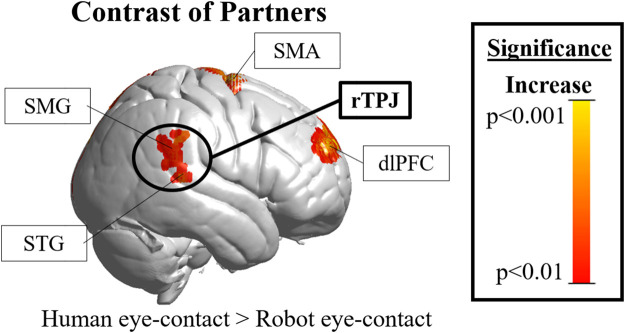
Cortical hemodynamic results. The rTPJ (black circle, the region of interest) was significantly more active during human than robot eye-contact (peak T-score = 2.8, uncorrected significance value *p* = 0.007, reaches FDR-corrected significance of *p* <0.05). rTPJ = right temporoparietal junction; DLPFC = right dorsolateral prefrontal cortex; SMA =supplementary motor area; SMG = supramarginal gyrus; STG = superior temporal gyrus.”

Figure 2 shows a colormap of eye-tracking behavioral results for subjects that had at least one valid eye-tracking data set per condition. The proportion of time per 3 s period spent looking at the eye-box of the partner is indicated by the color of the block with warmer colors indicating more hits and cooler indicating fewer. The figure shows that each participant engaged in more eye-contact during eye-contact periods of the task block than during no eye-contact periods. This demonstrates task compliance regardless of partner. Two participants appeared to make less eye-contact with their robot partner than they did with their human partner (subjects 2 and 4), though both still showed task compliance with the robot. In order to ensure that hemodynamic differences in processing the two partners were not confounded by the behavioral differences of these two subjects, contrasts were performed on the group both including (Figure 3, *n* = 15) and excluding the two subjects. Results were not meaningfully changed by their exclusion.

### Hemodynamic Results

Results in the right hemisphere of hemodynamic contrasts are shown in Figure 3, Supplementary Figure S1 and Table 1. The black circle indicates the rTPJ region of interest. Eye-contact with another human contrasted with baseline (Supplemenatry Figure S1, left) resulted in significant activity in the rTPJ (peak T-score = 4.27; *p* <0.05 FDR-corrected). ROI descriptive statistics: beta value x̄ = 5.12x10^−4^; σ = 9.31x10^−4^; 95% confidence interval: 4.87x10^−4^–5.38x10^−4^. Eye-contact with a robot contrasted with rest (Supplementary Figure S1, right) resulted in no significant activity in the rTPJ. ROI descriptive statistics: beta value x̄ = -4.25x10^−4^; σ = 0.002; 95% confidence interval: -4.81x10^−4^ — -3.69x10^−4^.

**TABLE 1:** Activity clusters in the right hemisphere.

	Human eye-contact > rest					
Anatomical name	Peak T-Score	*p*-value	X	Y	Z	Voxel #
R superior temporal gyrus (STG)	4.27	<0.001	70	−42	10	697
R occipital visual area II	3.97	<0.001	22	−98	8	275
R occipital visual area III	3.79	<0.001	0	−98	28	119
R dorsolateral prefrontal cortex (dlPFC)	2.84	0.007	28	56	28	46

	**Robot eye-contact > rest**					
Anatomical name	Peak T-Score	*p*-value	X	Y	Z	Voxel #
R occipital visual area II	3.02	0.005	12	−96	6	28
R occipital visual area II	4.53	<0.001	30	−96	6	778
R occipital visual area III	3.00	0.005	50	−68	−4	35
R premotor cortex	3.58	0.002	58	8	40	46

	**Human eye-contact > robot eye-contact**					
Anatomical name	Peak T-Score	*p*-value	X	Y	Z	Voxels #
R supramarginal gyrus (SMG)	2.80	0.007	68	−38	32	68
R dorsolateral prefrontal cortex (dlPFC)	3.96	<0.001	20	58	34	227
R supplementary motor area (SMA)	3.95	<0.001	20	−4	74	160

Statistics of hemodynamic results. The table shows statistics for positive clusters of significant fNIRS hemodynamic activity in the right hemisphere for three contrasts: human eye-contact > baseline (top); robot eye-contact > baseline (middle); and human eye-contact > robot eye-contact (bottom). (Column 1) the name of the cortical feature where the cluster is located. (Column 2) the maximum T statistic found within the cluster. (Column 3) the significance *p*-value, shown uncorrected. (Column 4–6) three dimensional coordinates in X, Y, and Z dimensions, given in Montreal Neurological Institute (MNI) space. X indicates right-left; Y indicates front-back; Z indicates up-down. (Column 7) the number of voxels in that cluster that reach significance. The superior temporal gyrus and supramarginal gyrus are the anatomical features of the temporoparietal junction (rTPJ). Analyses utilized a cluster size threshold of 20.

A comparison of the two partners (Figure 3) resulted in significant differences in the rTPJ (peak T-score: 2.8, *p* <0.05 FDR-corrected). Effect size: Cohen’s *d =* 0.5844. Exploratory whole brain analyses were also performed to identify other regions that might be of interest in future studies. Un-hypothesized significant differences were found in the right dorsolateral prefrontal cortex (rDLPFC) in a comparison of human eye-contact and rest (peak T-score: 3.96, *p* = 0.007, uncorrected) as well as human eye-contact compared to robot eye-contact (peak T-score = 2.84; *p* <0.001, uncorrected). A list of all regions that reached significance in the exploratory analyses can be found in Table 1.

## Conclusion and Discussion

The paradigm utilized here demonstrates how assessment of neural activity can give insight which may not be available through assessing behavior alone and offers an effective way to address some questions about naturalistic interaction between both humans and robots. Past attempts to apply fNIRS to human-robot interaction have explored its use as a signal transducer from human to robot as well as an evaluation tool (Kawaguchi et al., 2012; Solovey et al., 2012; Canning and Scheutz, 2013; Strait et al., 2014; Nuamah et al., 2019). This study builds on these by expanding data collection to the full superficial cortex and comparing neural processing during human-robot interaction to human-human interaction. It establishes a foundation for novel approaches to assess social robot design which may complement existing approaches (Gazzola et al., 2007; Sciutti et al., 2012a; Sciutti et al., 2012b).

As hypothesized, differences in rTPJ activity were found between processing of the human and robot partner, and these differences shed light on human social processing as well as Maki’s success in engaging it. While Maki’s simplified appearance was enough to control for low level visual features of making eye-contact with a human, as demonstrated by the lack of significant differences in occipital visual face processing areas (Dravida et al., 2019; Haxby et al., 2000; Ishai et al., 2005), Maki’s performance was not able to engage the rTPJ (Figure 3, black circle). This suggests that the combination of features that Maki shares with another human—superficial appearance, dynamic motion, coordinated behavior, physical embodiment (Wang and Rau, 2019), and co-presence (Li, 2015)—are not sufficient to engage the rTPJ. These results also give insight into Maki’s success in simulating a human partner: this robot, engaged as a novel partner with no preconceptions about abilities, and engaging in simulated eye-contact is not effective at engaging the naturalistic social processing network, and this difference was found despite comparable behavior with both partners.

These results inspire many questions: What in robot design is necessary and sufficient to engage the human rTPJ? Is it possible for Maki to engage it, and if so, what characteristics would do so? What impact would rTPJ engagement have on interpersonal relationship building with and recognition of Maki as “something like me” (Dautenhahn, 1995)? How does rTPJ engagement and any corresponding differences in impression and interaction relate to short- and long-term individual and public health, such as combatting loneliness?

Additional insight can be gleaned from the presented data: in addition to hypothesized rTPJ differences, the right dorsolateral prefrontal cortex (rDLPFC) also showed significant differences during human-human interaction and human-robot interaction, though these results were exploratory and thus do not reach FDR-corrected significance. The rDLPFC has been hypothesized to be critical for evaluating “motivational and emotional states and situational cues” (Forbes and Grafman, 2010), as well as monitoring for errors in predictions for social situations (Burke et al., 2010; Brown and Brüne, 2012) and implicit theory of mind (Molenberghs et al., 2016). This suggests that participants are spontaneously engaging in prediction of their human but not their robot-partners mind set, and are monitoring for differences in their expectations about their partner. RDLPFC activity has been tied to processing of eye-contact during human-human dialogue (Jiang et al., 2017) and to disengagement of attention from emotional faces (Sanchez et al., 2016). This suggests that candidate features to influence this system include subtle facial features meaningful for communication and reciprocation like eyebrows or saccades, or behavioral cues such as moving the gaze around the face during eye-contact. Activity in the rDLPFC has also been tied to strategizing in human-human but not human-computer competition (Piva et al., 2017). This suggests that top-down influences, such as preconceptions that people have of other people and of robots relating to agency, ability, or intention, are also important, and past research suggests that both bottom-up and top-down features are likely at play (Ghiglino et al., 2020a; Ghiglino et al., 2020b; Klapper et al., 2014). Lack of prior experience—which would create such preconceptions—may also be driving this difference.

The opportunities offered by the application of fNIRS to human-robot interaction are mutually beneficial to both social neuroscience and social robotics (Henschel et al., 2020). FNIRS to assess human-robot interaction can be used to interrogate whether robot interaction can incur naturalistic sociocognitive activity, how different elements of robot design impact that neurological processing, and how such differences relate to human behavior. It can also be used to parse the relationship between stimuli and human social processing. The data presented here offers a starting point for research to explore how the complex whole of appearance and behavior is brought to bear on social processing during direct eye-to-eye contact in human-human and human-robot interaction. Future studies may explore the impact of dynamic elements of robot design; subtle components of complex robot behavior; and pre-existing experience with or belief about the robot on human processing. This would give insight into the function of the human social processing system and answer the question of if it is possible for a robot to engage it. Processing during eye-contact should also be related to self-reported changes in impression of and engagement with the robot during various situations. Behavioral studies have shown that participants report greater engagement with a robot if it has made eye-contact with them prior to performance of a task (Kompatsiari et al., 2019). Whether these differences correlate with neural behavior remains to be seen. There are also other behaviors and brain regions that these techniques can be applied to. For instance, humans engage in anticipatory gaze when viewing goal directed robot behavior in a way more similar to viewing other humans than to viewing self-propelled objects (Sciutti et al., 2012b). Additionally, the rTPJ is just one system with implications on social perception of a robot. Motor resonance and the mirror neuron system are influenced by goals of behavior and show comparable engagement when human and robot behavior imply similar end goals, even when the kinematics of behavior are not similar (Gazzola et al., 2007). Future studies can explore how robot eye-contact behavior impacts this system and other such systems (Sciutti et al., 2012a). This approach may also be valuable for assessing nebulous but meaningful components of social interaction such as social engagement (Sidner et al., 2005; Corrigan et al., 2013; Salam and Chetouani, 2015; Devillers and Dubuisson Duplessis, 2017).

The promise of robots as tools for therapy and companionship (Belpaeme et al., 2018; Kawaguchi et al., 2012; Scassellati et al., 2018a; Tapus et al., 2007) also suggests that this approach can give valuable insight into the role of social processing in both health and disease. For instance, interaction with a social seal robot (Kawaguchi et al., 2012) has been linked to physiological improvements in neural firing patterns in dementia patients (Wada et al., 2005). This suggests a complex relationship between social interaction, social processing, and health, and points to the value of assessing human-robot interaction as a function of health. Social dysfunction is found in many different mental disorders, including autism spectrum disorder (ASD) and schizophrenia and social robots are already being used to better understand these populations. Social robots with simplified humanoid appearances have shown improvements in social functioning in patients with ASD (Pennisi et al., 2016; Ismail et al., 2019). This highlights how social robots can give insight which is not accessible otherwise as ASD patients regularly avoid eye-contact with other humans but seem to gravitate socially toward therapeutic robots. Conversely, patients with schizophrenia show higher amounts of variability than neurotypicals in perceiving social robots as intentional agents (Gray et al., 2011; Raffard et al., 2018) and show distinct and complex deficits in processing facial expressions in robots (Raffard et al., 2016; Cohen et al., 2017). Application of the paradigm used here to these clinical populations may thus be valuable for identifying how behaviors and expressions associated with eye-contact in robots and in people impact processing and exacerbate or improve dysfunction.

There are some notable limitations to this study. The sample size was small and some results are exploratory and do not reach FDR-corrected significance. As such, future studies should confirm these findings on a larger sample size. Additionally, fNIRS in its current iteration is unable to collect data from medial or subcortical regions. Past studies have shown that, for example, the medial prefrontal cortex is involved in aspects of social understanding (Mitchell et al., 2006; Ciaramidaro et al., 2007; Forbes and Grafman, 2010; Bara et al., 2011; Powell et al., 2018) and thus is hypothesized to be important to the paradigm utilized here. Such regions are important for gaining a comprehensive understanding of this system and thus some details of processing are being missed when using fNIRS.

## Data Availability Statement

The raw data supporting the conclusions of this article will be made available by the authors, without undue reservation.

## Ethics Statement

The studies involving human participants were reviewed and approved by Yale Human Research Protection Program. The patients/participants provided their written informed consent to participate in this study. Written informed consent was obtained from the individual in Figure 1 for the publication of potentially identifiable images included in this article.

## Author Contributions

MK, JH, and BS conceived of the idea. MK primarily developed and carried out the experiment and carried out data analysis. JH, AN, and XZ provided fNIRS, technical, and data analysis expertise as well as mentorship and guidance where needed. BS provided robotics expertise. MK along with JH wrote the manuscript.

## Conflict of Interest

The authors declare that the research was conducted in the absence of any commercial or financial relationships that could be construed as a potential conflict of interest.

## Supplementary Material

FIGURE S1 | **Cortical hemodynamic results**. Results of a contrast of task and rest blocks when partnered with the human **(left)** and the robot **(right)**. **(Left)** Significantly greater activity during human eye-contact was found in visual regions (V2 and V3) as well as the rTPJ. **(B)** Significantly greater activity during robot eye‐contact was found in visual regions. rTPJ = right temporoparietal junction; rDLPFC=dorsolateral prefrontal cortex; V2 & V3 = Visual Occipital Areas II and III.
