# Supplementary figures and images for: Comparison of Human Social Brain Activity During Eye-Contact With Another Human and a Humanoid Robot
Source: Front Robot AI. 2021 Jan 29;7:599581. doi: 10.3389/frobt.2020.599581 (PMC7879449; doi:10.3389/frobt.2020.599581)

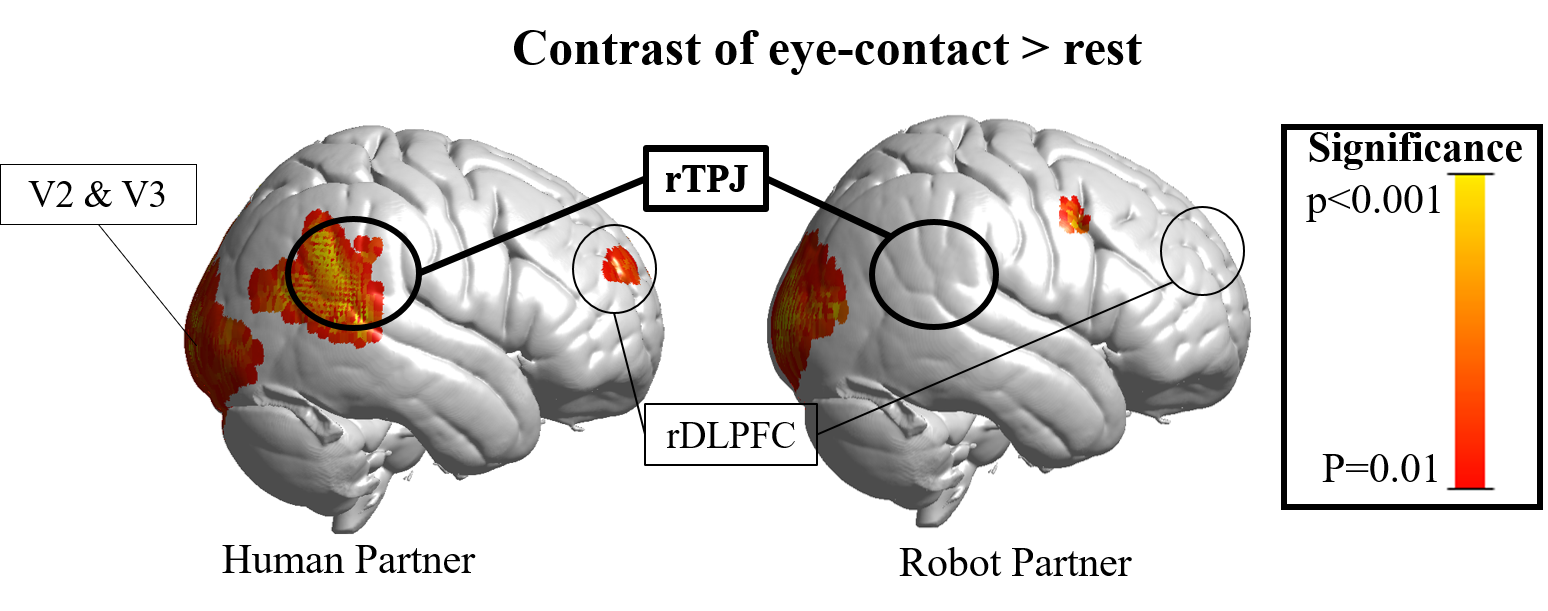

Supplement: Supplementary file 1 [file Image1.tif]
